# The Blood of the HIV-Infected Patients Contains κ-IgG, λ-IgG, and Bispecific κλ-IgG, Which Possess DNase and Amylolytic Activity

**DOI:** 10.3390/life12020304

**Published:** 2022-02-17

**Authors:** Anna Timofeeva, Sergey Sedykh, Lada Maksimenko, Tatyana Sedykh, Sergey Skudarnov, Tatyana Ostapova, Svetlana Yaschenko, Natalya Gashnikova, Georgy Nevinsky

**Affiliations:** 1 SB RAS Institute of Chemical Biology and Fundamental Medicine, 630090 Novosibirsk, Russia; sedyh@niboch.nsc.ru (S.S.); ta.al.gu@gmail.com (T.S.); nevinsky@niboch.nsc.ru (G.N.); 2 Faculty of Natural Sciences, Novosibirsk State University, 630090 Novosibirsk, Russia; 3 State Research Center of Virology and Biotechnology Vector, 630559 Koltsovo, Russia; maksimenko_lv@vector.nsc.ru (L.M.); ngash@vector.nsc.ru (N.G.); 4 Krasnoyarsk Regional Center for Prevention and Control of AIDS, 660049 Krasnoyarsk, Russia; gl_vrach@aids.krsn.ru (S.S.); ostapova@aids.krsn.ru (T.O.); yashchenko@aids.krsn.ru (S.Y.)

**Keywords:** HIV, bispecific antibodies, IgG, immunoglobulin G, catalytic activity, abzymes, DNAse activity, DNA hydrolysis, amylolytic activity, ELISA

## Abstract

Though hundreds of thousands of papers are currently being published on HIV/AIDS, only tens of hundreds of them are devoted to the antibodies generated during the disease. Most of these papers discuss antibodies in HIV/AIDS as a diagnostic tool, and some articles describe neutralizing antibodies as a promising treatment. In this paper, we used affinity chromatography and ELISA to isolate natural IgG from the blood of 26 HIV-infected patients. IgG preparations were separated into the subfractions containing different types of light chains, and catalytic activities of subfractions were analyzed. Here, we show for the first time that the blood of HIV patients contains ~20% of bispecific κλ-IgG, presented with all IgG subclasses. Analysis of DNA-hydrolyzing and amylolytic activity show that most IgG preparations and subfractions are catalytically active. Our results expand the possible biological functions of natural IgG in HIV infection.

## 1. Introduction

HIV infection is a disease caused by the human immunodeficiency virus (HIV) [1,2], which may be slowly progressive or instead with a dramatic manifestation of clinical symptoms. HIV infects cells of the immune system with CD4 receptors on their surface: T-helpers, monocytes, macrophages, Langerhans cells [3], dendritic cells, and microglia cells [4]. As a result, the work of the immune system is suppressed; acquired immune deficiency syndrome (AIDS) develops; the patient’s body loses its ability to defend itself against infections and tumors; and opportunistic diseases, not typical for people with normal immune status, arise [5,6,7]. The presence of autoimmune phenomena and the appearance of autoantibodies in HIV/AIDS and some other viral infections may be associated with the activation of polyclonal B-cells, molecular mimicry between viral or microbial antigens and host antigens [8,9], abnormal expression of immunoregulatory molecules, and anti-idiotypic network [10,11]. 

Activating B-lymphocytes in HIV-infected patients results in the production of antibodies (Abs) to viral components and autoantibodies (autoAbs) to many different components of human cells [11]. In addition, as other authors have shown previously, IgG or IgM of HIV/AIDS patients hydrolyze DNA [12], myelin basic protein [13], histones [14,15,16,17], HIV integrase [18,19,20], and HIV reverse transcriptase [21]. Data on the analysis of catalytic antibodies in HIV-infected patients are summarized in the review [22].

DNA and anti-DNA Abs at elevated concentrations, which are believed to be produced due to cell apoptosis, are detected in the blood of patients with certain autoimmune diseases [23,24]. Of all the known pathologies, only systemic lupus erythematosus (SLE) is generally considered to be associated with auto immunization of patients with DNA; sera from SLE patients usually contain high concentrations of DNA [25,26].

The exact biological function of DNA-hydrolyzing Abs is currently unknown; according to published data, it can be either beneficial or harmful for the patients. However, serum from patients with several diseases has been shown to contain DNA and anti-DNA Abs [27] and RNA and anti-RNA Abs [28,29]. Anti-DNA Abs are found in the blood serum of healthy mammals, but their titers vary significantly and are usually low [27].

It has been shown that DNA-hydrolyzing Abs are one of the earliest markers of autoimmune pathologies. For example, it has been demonstrated that such DNA-hydrolyzing Abs exhibit cytotoxicity, cause fragmentation of nuclear DNA, and may induce cell apoptosis [30].

Abs hydrolyzing oligosaccharides were isolated from the blood [31] and cerebrospinal fluid [32] of patients with multiple sclerosis and the milk of healthy women [33]. Their possible biological activity is also insufficiently studied.

Earlier, from the blood of healthy donors, placenta, and milk obtained from healthy women, we isolated IgG simultaneously containing two types of light chains (κλ-IgG). The relative content of such bispecific IgG significantly varies in the blood (9%), milk (up to 54%), and placenta extract (15%). Could such Abs be markers of immunodeficiency or autoimmune and viral diseases? Are the various IgG subfractions of the blood of HIV-infected people catalytically active? We decided to test these questions on the patients who have not received antiretroviral therapy.

## 2. Materials and Methods

### 2.1. HIV-Infected Patients: Clinical Parameters and Sample Collection

Peripheral blood plasma samples were collected from HIV-infected patients living in Krasnoyarsk, who sought assistance in Krasnoyarsk Regional Center for Prevention and Control of AIDS. Plasma was separated within 3 h after collection and frozen at −80 °C for further use. Blood samples were linked with clinical and demographic data through coded ID numbers, according to medical ethical requirements in Russia. Characteristics for patients included their gender, age, most probable route of transmission, dates of the first positive (and the last negative) tests for HIV, drug use, viral load, and CD4 cell count. Twenty-six HIV-infected patients were selected, of which twenty-two patients were at the third stage of the disease; patients on the 4A, 2A, and 2B stages were also analyzed (see Table 1 for patients’ characteristics). No single patient took any antiretroviral therapy; the prevailing sub-subtype of the HIV was A6, and viral load ranged from 10,500 to 175,000 copies/mL. The protocol of blood sampling was confirmed by the local human ethics committee (Novosibirsk State Medical University, Novosibirsk, Russia; number 105-HIV; 07. 2010).

### 2.2. Antibody Purification and Analysis

Electrophoretically and immunologically homogeneous IgGs from the blood plasma of each patient were obtained by affinity chromatography on protein-G-Sepharose, similarly to [34,35,36]. The absence of viral RNA in the obtained IgG preparations was determined by real-time RT-PCR (RealBest HBV/HCV/HIV PCR, Russia). The isolation of IgG was carried out at the State Research Center of Virology and Biotechnology “Vector” using 1 mL protein G columns (GE Life Sciences, New York, NY, USA).

Electrophoretic homogeneity of the IgG preparations was shown using a 4–15% gradient SDS-PAGE followed by Coomassie blue staining. Each IgG preparation was assayed in the presence and absence of 40 mM DTT before boiling for 2 min. Incubation of IgG with dithiothreitol and subsequent boiling for 2 min leads to the reduction of disulfide bonds and reveals two bands corresponding to the heavy (~50 kDa) and light (~25 kDa) chains. For the Western blot analysis, we used a mix of secondary goat anti-human-L-chain HRP conjugates (A7164 and A5175, Sigma, St. Louis, MO, USA). IgG preparations were considered electrophoretically homogeneous if the Coomassie staining showed a single band corresponding to IgG with no additional bands. For the protein molecular mass standards, we used commercial recombinant proteins expressed in prokaryotes that did not have such post-translational modifications, such as glycosylation. On the contrary, natural IgG isolated from human blood had multiple glycosylation sited. Since that, the visible molecular mass of the band corresponding to IgG generally increased to 150 kDa, which differs from the standards described in immunology textbooks.

To obtain IgG preparations containing different types of light chains (κ—kappa and λ—lambda), the fraction of total IgG was sequentially applied to KappaSelect and LambdaSelect columns using GE Akta Start (GE Life Sciences, New York, NY, USA) according to the method previously developed in our laboratory [37]. Columns were washed with TBST (Tris-buffered saline containing 0.1% Triton) and TBS (Tris-buffered saline, containing 150 mM NaCl and 20 mM Tris, pH 7.5) to zero optical absorption on A_280_. Proteins were eluted at 0.8 mL/min with 0.1 M glycine pH 2.6; the protein yield was monitored by changing the optical density of the eluate at λ = 280 nm. Eluted κ-IgG and λ-IgG were dialyzed against 20 mM Tris-HCl pH 7.5. Preparations of κ-IgG eluted from the KappaSelect with acid buffer were loaded onto LambdaSelect, and washed with TBS, TBST, and TBS to zero absorbance on A_280_. Bispecific κλ-IgG was eluted with 0.1 M glycine pH 2.6. Similarly, λ-IgG was loaded onto KappaSelect, washed as described above, and bispecific κλ-IgG was eluted with 0.1 M glycine, pH 2.6.

Subfractions of κκ-IgG, λλ-IgG, and bispecific κλ-IgG obtained by affinity chromatography of KappaSelect and LambdaSelect columns were analyzed with the enzyme-linked immunosorbent assay (ELISA).

### 2.3. ELISA of IgG, κ-IgG, λ-IgG and κλ-IgG

Aliquots of 100 μL primary Abs (anti-kappa K-4377, anti-lambda L-6522, both from Sigma, St. Louis, MO, USA), with a final concentration of 1 μg/mL, were dissolved in 0.1 M carbonate–bicarbonate buffer (0.1 M NaHCO_3_, 0.1 M Na_2_CO_3_), and incubated overnight at 4 °C in wells of ELISA strips with a high binding surface (Corning, Tewksbury, MA, USA). Surface of wells was blocked by adding 200 μL of 5% skimmed milk powder (Applichem, Darmstadt, Germany) in TBST, incubated for 60 min at 37 °C on a shaker incubator (Biosan, Riga, Latvia). Blocking solutions were removed out of the wells and washed with 200 μL of TBST three times each. Preparations of analyzed IgG with 0.1 mg/mL concentration were diluted ten times with TBS and added in 100 μL per well. ELISA plates were incubated for 1 h at 37 °C on a shaker incubator. Aliquotes of 100 μL of secondary Abs conjugated to horseradish peroxidase were diluted according to the manufacturer’s instructions (anti-kappa A-7164 20,000 times, anti-lambda A-5175 20,000 times, both from Sigma, St. Louis, MO, USA) in TBST and incubated for 1 h at 37 °C shaker incubator. Solutions were removed out of the wells and washed with 200 μL of TBST three times. Then, the wells were filled with 100 μL of the substrate (5 mL of citrate-phosphate buffer pH 5, 12 μL of 30% hydrogen peroxide, 5 mL of distilled water, 715 μL of 5 mg/mL TMB solution in DMSO). After 15 min, the reaction was stopped by adding 50 μL of 1 M sulfuric acid. Optical density was measured on a Multiskan FC spectrophotometer (Thermo Scientific, Waltham, MA, USA) in a two-wave mode, with a primary filter of 450 nm and a reference filter of 620 nm. Results were presented as the average of a series of three independent experiments. 

Concentrations of IgG1–IgG4 in IgG preparations were determined with 96-well plates containing immobilized Abs against IgG1–IgG4 subclasses. Aliquotes of 100 μL of IgG with a concentration of 0.1 mg/mL, diluted 50 times with TBS, were incubated for 30 min at 37 °C. Then, 100 μL of anti-human-IgG conjugated with horseradish peroxidase (Vector-Best, Novosibirsk, Russia) was added to the wells, and solutions were incubated for 30 min at 37 °C and washed three times with TBST. One hundred microliters of solution 3,3’,5,5’-tetramethylbenzidine (TMB, Dia-M, Moscow, Russia) was added to wells; the plates were incubated for 10 min in the dark, and the reaction was stopped by adding 100 μL of 1 M sulfuric acid. The optical density was measured, as described above.

The concentrations of IgG1–IgG4 were determined using standard samples; the calibration graph was plotted in Microsoft Excel. Results were presented as the average value in a series of three wells for each sample and each IgG subclass.

### 2.4. Analysis of DNase Activity

DNA hydrolysis activity was analyzed using supercoiled pBluescript DNA as previously described [34,38]. Furthermore, 20 μL of reaction mixture contained 18 μg/mL (6.1 nM) of supercoiled pBluescript DNA, 5.0 mM MgCl_2_, 1.0 mM EDTA, 20.0 mM Tris-HCl, pH 7.5, and IgG in final concentration of 0.01 mg/mL. Samples were incubated for 48 h at 37 °C. Relative amounts of DNA in the supercoiled, linear, and relaxed plasmid bands were analyzed using ImageQuant v.5.2 (Molecular Dynamics, Los Angeles, CA, USA). The activity of IgG preparations was determined by the decrease in the percentage of dsDNA converted from the original supercoiled form to relaxed and linear forms. Control samples did not contain Abs and were incubated for up to 120 h to prove that non-specific hydrolysis does not occur. All measurements were carried out in linear regions of hydrolysis (15–40% of DNA hydrolysis), and the complete transition of the supercoiled plasmid to the hydrolyzed form was taken as 100% activity.

Methods of in situ DNAse activity were the most reliable method to prove the intrinsic catalytic activity of Abs. In short, the preparations of IgG were separated in SDS-PAGE in gel, containing 5 µg/mL of calf thymus DNA. After the electrophoresis, the gel was washed in 0.1% Triton X-100 for 1 h and in TBS for 3 h, and then incubated in TBS containing 4 mM MgCl_2_ for 3 days. Gel was stained with 0.5 μg/mL of EtBr for 1 h, and the products of DNA hydrolysis were visualized on Gel Doc XR+ (Bio-Rad, Hercules, CA, USA) as unstained with EtBr zones, corresponding to the bands of IgG after Coomassie blue R-250 staining. The absence of zones unstained with EtBr indicates that DNase activity is an intrinsic property of protein corresponding to the IgG on molecular mass and is not due to impurities of any canonical nucleases (none of the nucleases with such molecular mass are described).

### 2.5. Analysis of Amylolytic Activity

The amylolytic activity of IgG preparations was measured in 10 μL of reaction mixture containing 20 mM Tris-HCl, pH 7.5, and 5.0 mM maltoheptaose (M7753, Sigma, St. Louis, MO, USA). The reaction was started by adding 0.01 mg/mL of IgG preparation to the mixture. Samples were incubated at 37 °C for 48 h. Control samples were incubated up to 120 h without Abs to show that non-specific hydrolysis does not occur. Two-microliter aliquots of the reaction mixture were applied to Silica gel 60 TLC plates (Sigma, St. Louis, MO, USA). Products of maltoheptaose hydrolysis were separated with thin-layer chromatography in the system acetic acid:water:butanol mixture at a ratio of 1:1:3, respectively. Plates were dried, treated with a mixture of sulfuric acid and propanol-2 at a ratio of 1:7, dried, and heated to 110 °C to visualize the hydrolysis products. Results were recorded using an office scanner. The complete hydrolysis of maltoheptaose was taken as 100% activity. Results of hydrolysis were analyzed using ImageQuant v.5.2.

Similar to the in situ DNase activity, the amylolytic activity was proven to be an intrinsic activity of IgG isolated from the blood of HIV-infected patients using eluates from the gel after SDS-PAGE. In short, the gel strip corresponding to the sample of IgG was washed from SDS and other buffer components in 0.1% Triton X-100 for 1 h, then in TBS for 3 h, sliced into tiny pieces with a 1–2 mm width, and eluted in separate test tubes with TBS. Aliquotes of eluates were used to measure the amylolytic activity as described above. The absence of maltoheptaose hydrolysis in fragments other than IgG-containing gel slices gave us evidence of the intrinsic nature of the amylolytic activity of IgGs. 

### 2.6. Statistical Analysis

We used correlation analysis (the non-parametric Spearman rank method) of biochemical characteristics of Abs and various clinical data of patients. Experimental results were presented as the mean value ± standard deviation of three independent experiments for each IgG sample. Errors of measurement did not exceed 5%. The correlation between the samples was assessed using the Pearson test. Two-sided *p* < 0.05 was considered statistically significant.

## 3. Results

### 3.1. Characteristics of Patients: Isolation of Antibodies

Electrophoretically and immunologically homogeneous IgG preparations were isolated from the blood plasma of 26 HIV-infected patients in the Department of Retroviruses, State Research Center of Virology and Biotechnology “Vector” (Koltsovo, Russia). None of the patients took antiretroviral therapy; there were no pregnant women among the donors. The predominant sub-subtype of the virus was A6, with a viral load from 10,500 to 175,000 copies per ml. Patients’ characteristics are given in Table 1.

The isolation of IgG using affinity chromatography on protein-G-Sepharose was performed according to our previously published protocols [13,39,40]. The absence of virus RNA in the obtained IgG preparations was confirmed by real-time RT-PCR, as in [41,42]. 

**Figure 1:**
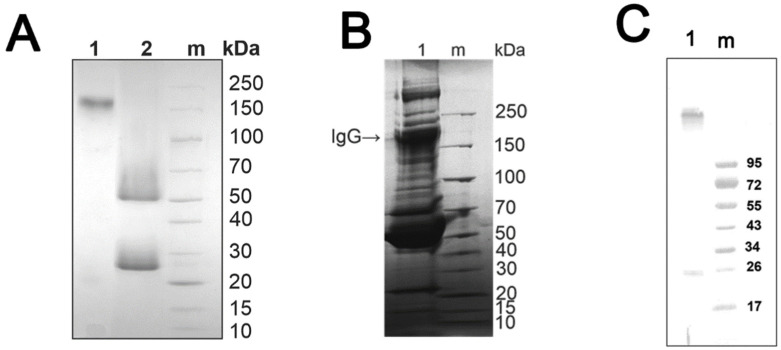
SDS-PAGE (**A**,**B**) and Western blot (**C**) analysis of IgG homogeneity. SDS-PAGE in 4–18% gradient polyacrylamide gel. Line 1—intact IgG (**A**,**C**), 0.5 μL of human blood plasma of healthy donor (**B**); line 2—IgG incubated with 40 mM DTT at 100 °C; line m—protein molecular weight markers (**A**,**B**—26630, Thermo Scientific, Waltham, MA, USA); **C**—SM0671 pre-stained markers, Fermentas, Vilnius, Lithuania).

SDS-PAGE (Figure 1A,B) and Western blot (Figure 1C) showed the electrophoretic homogeneity of all IgG preparations. Only a single band with a molecular mass slightly exceeding 150 kDa was detected in the region typical for IgG (Figure 1A). A band of >150 kDa molecular mass is also one of the major bands in preparations of human blood plasma (Figure 1B). Western blot analysis showed that the IgG preparations contain a major band of IgG and a minor band of free light chain (Figure 1C). The original WB images for Figure 1 are showing in Figure S1.

### 3.2. Isolation and Analysis of κκ-IgG, λλ-IgG and κλ-IgG Subfractions

**Figure 2:**
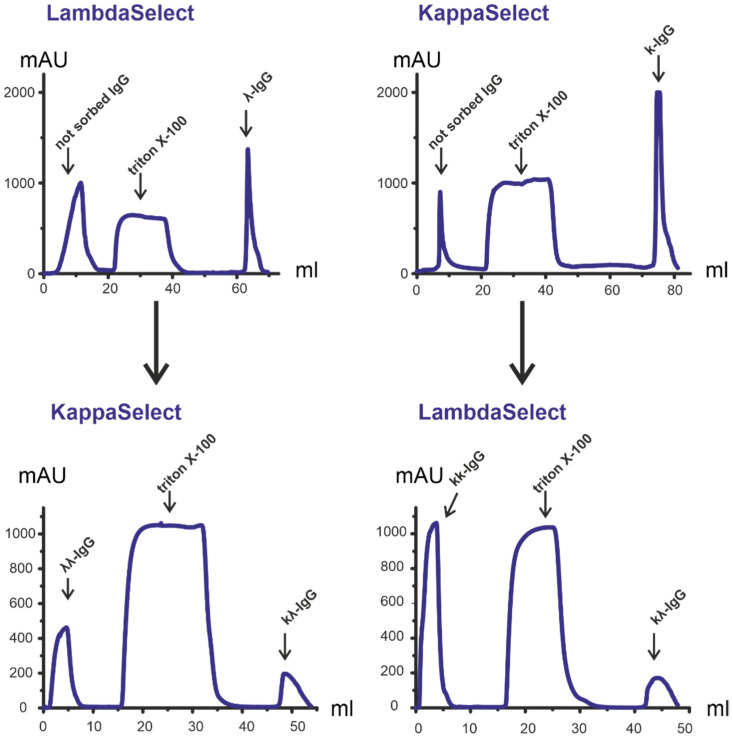
Affinity chromatography of total IgG preparation isolated from HIV-infected patients on KappaSelect and LambdaSelect columns allowed us to obtain three subfractions: κκ-IgG, λλ-IgG, and κλ-IgG.

We developed and published a method for the isolation of bispecific antibodies (bsAbs) from human milk [43], blood [37], and placenta [44]. Here, the preparations of κκ-IgG, λλ-IgG, and bispecific κλ-IgG Abs were isolated from IgG using affinity chromatography on columns which bind explicitly human light chains of the kappa type (KappaSelect, GE Life Sciences) and lambda type (LambdaSelect, GE Life Sciences). KappaSelect bound κκ- and κλ -IgG, while LambdaSelect bound λλ- and κλ-IgG. IgG fraction with an affinity for KappaSelect was rechromatographed on LambdaSelect and vice versa (Figure 2). The κλ-IgG subfraction, with an affinity for KappaSelect, also binds to LambdaSelect and vice versa. Thus, κλ-IgG subfractions were eluted with acid buffer both from LambdaSelect and KappaSelect on repeated chromatography, while κκ-IgG and λλ-IgG were obtained in-flow during second chromatography. The exact mechanism generation of κλ-IgG in the blood is unknown, but we suppose that these bsAbs are generated due to the Fab arms exchange described in [45,46]. However, at present, the mechanism of this process is described in detail only for IgG4 [47].

Three subfractions of Abs with different combinations of light chains were obtained from the mixture of ten aliquots (1 mg each) of IgG isolated from the blood of HIV-infected patients: κκ-IgG, λλ-IgG, and κλ-IgG (Figure 2). These samples were representative (5 + 5 man and women; with average values of age, viral load, and CD4 count close to the mean values) of the whole sample set of 26 patients. The peak integration of the chromatography profiles in the Unicorn (GE Life Sciences, New York, NY, USA) software made it possible to estimate the relative content of κκ-IgG, λλ-IgG, and κλ-IgG, which amounts were to 53.7%, 26.1%, and 20.2%, respectively. As previously established, the content of kappa light chains containing IgG in the blood of healthy donors is approximately twice as high as that of lambda-IgG; therefore, the amount of κκ-IgG and λλ-IgG corresponds with the data published in the literature [48]. It is not easy to interpret the amount of bispecific κλ-IgG which is comparable to the amount of λλ-IgG and twice as high as that in the blood of healthy human donors, i.e., ~9%, according to [37], and higher than in human placenta, i.e., 15% [44]. However, human milk contains much more bispecific κλ-IgG, i.e., up to 54% [43]. In the future, we plan to isolate κκ-IgG, λλ-IgG, and κλ-IgG from individual blood samples of HIV-infected patients, which will make it possible to determine the content of these subfractions in individual patients. 

**Figure 3:**
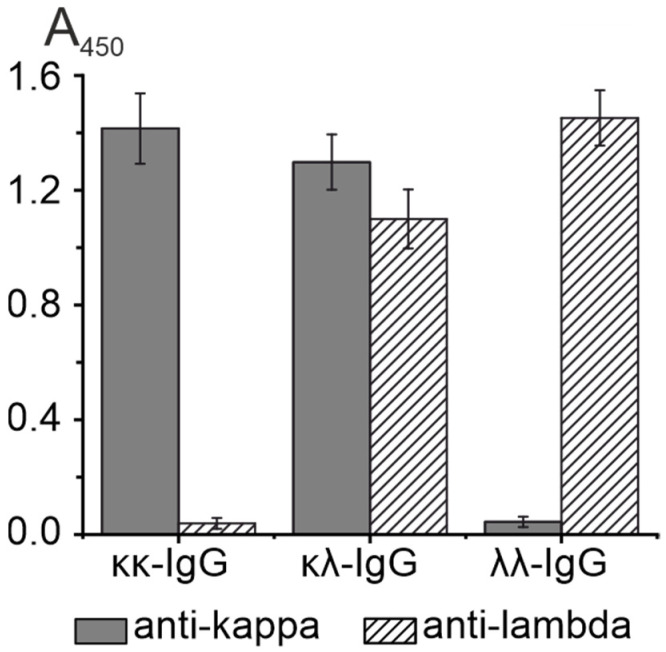
Double-sandwich ELISA of κκ-IgG, λλ-IgG, and κλ-IgG subfractions. A450 values are shown as the mean absorbance in three independent ELISA experiments. Error bars indicate the SD.

We used the double-sandwich ELISA to confirm that the isolated κκ-IgG, λλ-IgG, and κλ-IgG subfractions which contained the exact combinations of light chains. For example, they were not a mixture of κκ-IgG and λλ-IgG, like in the case of κλ-IgG. Primary anti-κ light chain and/or anti-λ light chain Abs were sorbed in the wells of the ELISA plates. Then, the subfractions of κκ-IgG, λλ-IgG, and κλ-IgG were added to the wells, respectively. After this, the anti-κ light chain or anti-λ light chain HRP conjugates were added for the detection. We observed a positive ELISA signal for κκ-IgG only when using a combination of primary anti-κ light chain and anti-κ light chain HRP conjugates. Similarly, for λλ-IgG, a positive signal was observed only when using the anti-λ light chain primary Abs and HRP conjugates. In the case of κλ-IgG, we observed a positive signal when using both combinations of primary anti-κ light chain Abs and anti-λ light chain HRP conjugates, and anti-λ light chain Abs and anti-κ light chain HRP conjugates. These results, presented in Figure 3 and Table S1, prove that we obtained κκ-Ig, λλ-IgG, and bispecific κλ-IgG without any co-isolating impurities.

Four IgG subclasses are known in human IgG, which have more than 90% similar sequence, but significantly differ in functions. For example, the response of IgG to bacterial infection is mainly associated with IgG2, while viral infections usually induce the production of IgG1 and IgG3 [49]. IgG4 is produced in the case of chronic antigen immunization [50]. The relative content of Abs of different subclasses in the blood serum of healthy donors varies significantly: IgG1 (34–87%), IgG2 (5–56%), IgG3 (0.5–12%), and IgG4 (7–12%) [37]. As mentioned above, the detailed mechanism of bispecific IgG generation is described only for IgG4 [47]. Therefore, it was interesting to analyze which subclasses of IgG represent the subfractions of κκ-IgG, λλ-IgG, and κλ-IgG.

**Figure 4:**
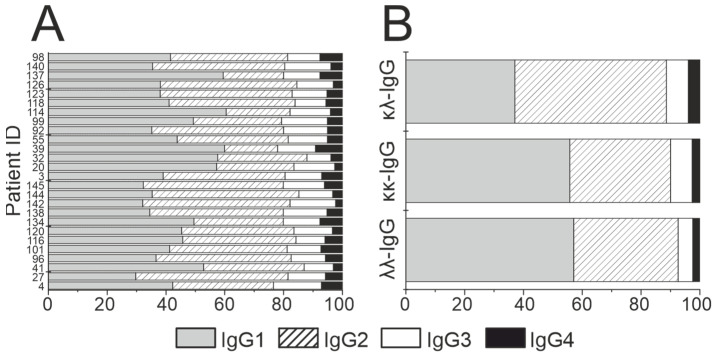
Double-sandwich ELISA analysis of IgG1–IgG4 subclasses among the IgG isolated from the blood of HIV-infected patients (**A**) and among the κκ-IgG, λλ-IgG and κλ-IgG subfractions isolated from total preparations of IgG across 10 patients (**B**).

The contribution of IgG1–4 subclasses to the immune response in HIV infection was analyzed. Individual IgG preparations were examined: IgG1 prevailed in 15 patients (50.4%), while the IgG2 subclass (32.1%) was the majority in other patients. Three patients at stage 4A and two patients at stage 3 had a significant predominance of IgG1 (60.0 ± 0.1%) > IgG2 (20.1 ± 2.1); the remaining patients contained IgG2 as a major subclass (46.7%) > IgG1 (35.1%). In all patients, the IgG3 content (12.8%) exceeded IgG4 (5.3%); results are presented in Figure 4A. Raw data are given in Table S2. Figure 4A shows that each patient is characterized by an individual ratio of IgG1–4 subclasses, which is consistent with the literature data.

IgG1–4 subclasses were tested in the κκ-IgG, λλ-IgG, and κλ-IgG subfractions (Figure 4B). The contents of κκ-IgG subclasses were IgG1 (55.8%) > IgG2 (34.2%) > IgG3 (7.4%) > IgG4 (2.6%); for λλ-IgG, they were IgG1 (57.1%) > IgG2 (35.5%) > IgG3 (5.0%) > IgG4 (2.3%); and for bispecific κλ-IgG, they were IgG2 (51.5%) > IgG1 (37.1%) > IgG3 (7.5%) > IgG4 (3.9%). Thus, the κλ-IgG was presented with all IgG1–4 subclasses (as in the case of the IgG of human blood, placenta, and milk [37,44,51]). Moreover, IgG1 prevailed in the κκ-IgG and λλ-IgG subfractions, and IgG2 in case of κλ-IgG subfractions. Raw data are given in Table S3.

### 3.3. DNase and Amylolytic Activities

**Figure 5:**
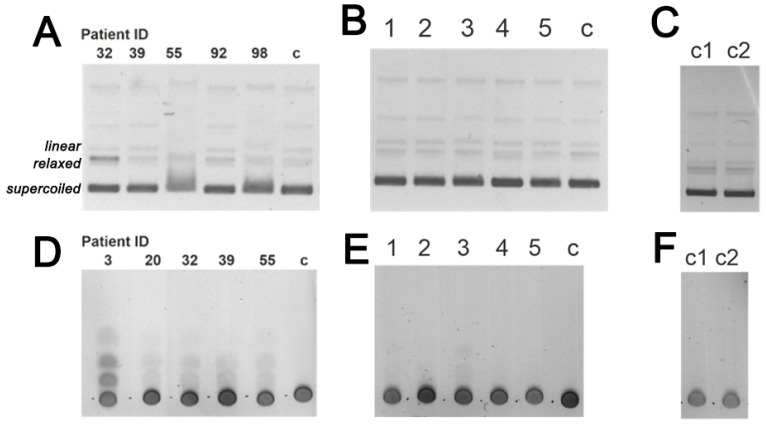
DNase and amylolytic activity of IgG were analyzed for preparation isolated from HIV (**A**,**D**) and healthy patients (**B**,**E**), time of incubation: 24 h (**A**) or 48 h (**D**), 37 °C. Control samples (**C**,**F**) were incubated without IgG for 72 h (c1) or 120 h (c2), 37 °C. Hydrolysis products were analyzed by agarose electrophoresis (**A**–**C**) and TLC (**D**–**F**). Final concentration of maltoheptaose—5 mM (**A**–**C**), of pBluescript—9.6 μg/mL, IgG—0.01 mg/mL (**A**,**B**), 0.05 mg/mL (**D**,**E**); c—control sample, incubation without Abs.

It is known that the blood of HIV-infected patients contains catalytic Abs, hydrolyzing DNA [12]; however, the amylolytic activity of HIV IgG has never been shown. Here, we show the analysis of DNase and amylolytic activity of IgG for each of the 26 patients. It was revealed that most of the patients’ IgG hydrolyze supercoiled plasmid DNA and maltoheptose, which corresponds to the data previously obtained in [30]. The level of catalytic activity is individual for each of the patients. Figure 5 shows an example of products of DNA hydrolysis (Figure 5A) using agarose gel electrophoresis and maltoheptaose hydrolysis (Figure 5D) using TLC for IgG preparations isolated from the blood of five patients. The level of DNase activity was measured as a conversion of the supercoiled form of DNA in linear and relaxed due to the generation of single-stranded or double-stranded breaks in plasmid DNA. Similarly, the percent of amylolytic activity was established through the generation of lower molecular weight products of oligomeric maltoheptaose. The control samples (Figure 5B,E), containing IgG isolated from five healthy donors, were incubated for 48 h. Other control samples (c1, c2) did not contain any IgG samples (Figure 5C,F) and were incubated for 72 and 120 h, respectively. The original images for Figure 5 are showing in Figure S2.

Using the conditions of Michaelis–Menten kinetics, it is possible to analyze early stages of IgG-mediated DNA hydrolysis (for example, see Figure 5A—patient IDs: 32 and 39), but some IgG preparations in the same conditions possess more than single-stranded or double-stranded break, and shorter fragments are visible (see Figure 5A—patient ID: 55). Similarly, several products of the maltoheptaose hydrolysis can be seen in Figure 5D.

**Figure A1:**
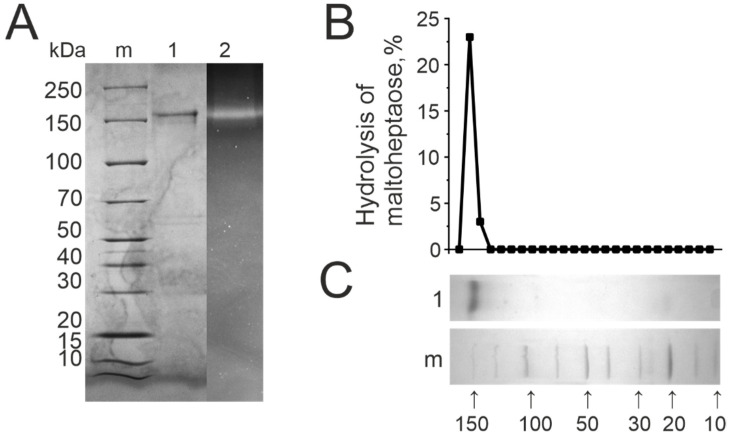
Analysis of IgG DNase (**A**) and amylolytic activity (**B**) after SDS-PAGE (**A**,**C**). Line 1—intact IgG, isolated from the blood of HIV-infected patients. Line 2—in situ analysis of DNase activity by SDS-PAGE with co-polymerized calf thymus DNA, staining with EtBr, visualization in UV. m—marker of protein molecular masses.

Analysis of catalytic Abs needs evidence that DNAse and amylolytic activity are intrinsic properties of IgG. Other methods that provide such proof include in situ DNase activity (see Figure A1A in Appendix A) and analysis of the amylolytic activity of eluates obtained from the gel slices after SDS-PAGE (Figure A1B,C in Appendix A). 

**Figure 6:**
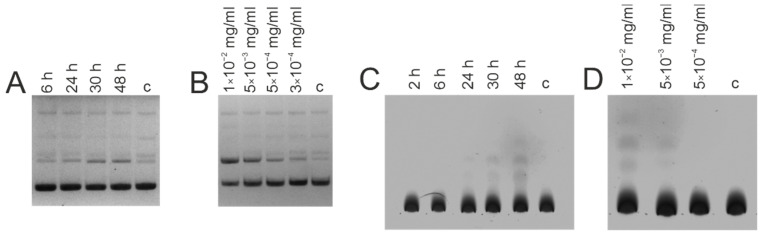
Time (**A**,**C**) and concentration (**B**,**D**) dependency of DNase (**A**,**B**) and amylolytic (**C**,**D**) activity of IgG isolated from the blood serum of HIV-infected patient ID 55. Final concentration of pBluescript DNA—9.6 μg/mL, maltoheptaose—5 mM, IgG—0.01 mg/mL (**A**), 0.05 mg/mL (**C**), presented in the figure (**B**,**D**); time of incubation—48 h (**B**,**D**), presented on the figure (**A**,**C**); c—control sample, incubation without Abs.

Other essential features of enzyme-like biocatalytic processes include the time and concentration dependences. For example, Figure 6 shows the results of analysis of DNase and amylolytic activity of IgG patient ID 55 in various time intervals (from 6 to 48 h and 2 to 48 h, respectively—Figure 6A,C), and final concentrations of IgG (from 0.0003 or 0.0005 to 0.01 mg/mL, respectively—Figure 6B,D). In Figure 6A,C, one can see an increase in the level of hydrolysis of DNA and maltoheptaose comparable with controls (6 h for DNase activity, 2 and 6 h for amylolytic activity, respectively) and an accumulation of products in 30 and 48 h for both activities which are visible to the naked eye. Figure 6B,D show a concentration-dependent decrease in activity, from visible to the naked eye at 0.01 mg/mL of IgG to indistinguishable from controls at 0.3–0.5 μg/mL. 

**Figure A2:**
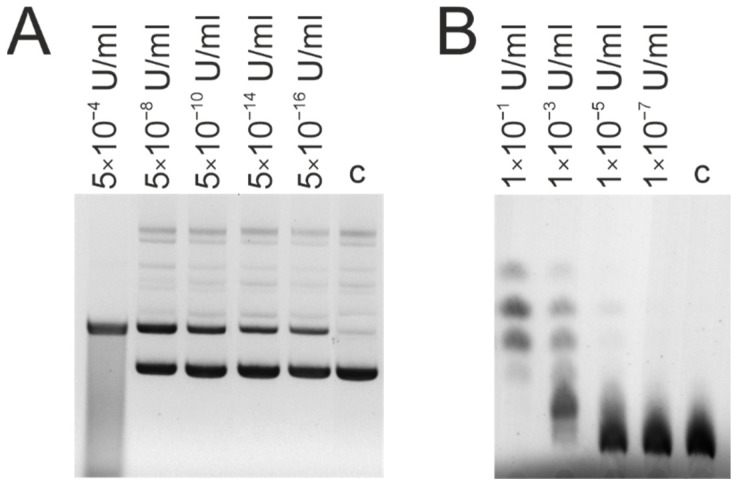
Hydrolysis of pBluescript plasmid DNA (**A**) and maltoheptaose (**B**) by DNase I (**A**) and *Bacillus *sp. α-amylase (**B**) at various final concentrations after incubation for 48 h at 37 °C; c—control samples incubated without enzymes. One unit of enzyme activity of DNase I according to the supplier instruction completely degrades 1 μg of plasmid DNA in 10 min at 37 °C (EN0525, Thermo Waltham MA, USA); 1 U of α-amylase from Bacillus sp. (A6380, Sigma, St. Louis, MO, USA) liberates 1 mg of maltose from starch in 3 min at pH 6.9 at 20 °C.

**Figure A3:**
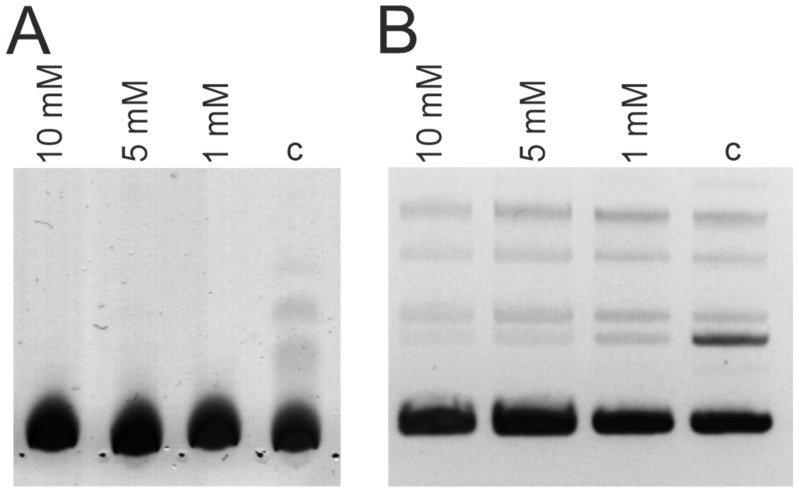
Inhibition of IgG-possessed hydrolysis of maltoheptaose (**A**) and pBluescript DNA (**B**) by EDTA (1–10 mM); final concentration of patient ID 55 IgGs—0.05 mg/mL; time of incubation—48 h, 37 °C; c—control samples incubated without EDTA.

We used the commercial DNase I and α-amylase preparations as positive controls of DNA and oligosaccharide hydrolysis (see Figure A2 in Appendix A) which show that, depending on the patient ID, specific DNase activity of IgG varies from 10^−19^ to 10^−13^ U to 1 mg of IgG, and amylolytic activity ranges from 10^−9^ to 10^−8^ U/mg of Abs (see Table A1). Most of the DNA-hydrolyzing enzymes (and catalytic Abs) described in the literature are metal-dependent, so EDTA is one of the common non-specific inhibitors of such enzymes. Similarly, the activity of α-amylase and other oligosaccharide hydrolyzing enzymes is inhibited with EDTA. Since that, we used EDTA in concentrations 1 to 10 mM to show the inhibition of these catalytic activities in patient ID 55 IgG preparations (see Figure A3).

**Figure 7:**
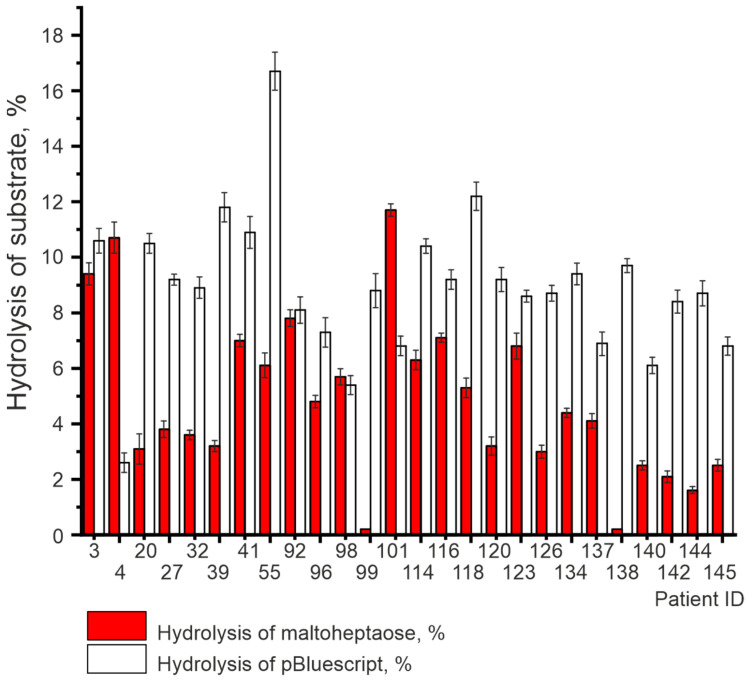
DNA-hydrolyzing and amylolytic activity of IgG isolated from the blood of HIV-infected patients. Complete hydrolysis of substrates in 24 h with Abs at 0.01 mg/mL was taken as 100%. Error bars indicate the SD.

In Figure 7, we present the summary data of DNA hydrolyzing and amylolytic activities. Raw data for these catalytic activities are given in Table S4. 

**Figure 8:**
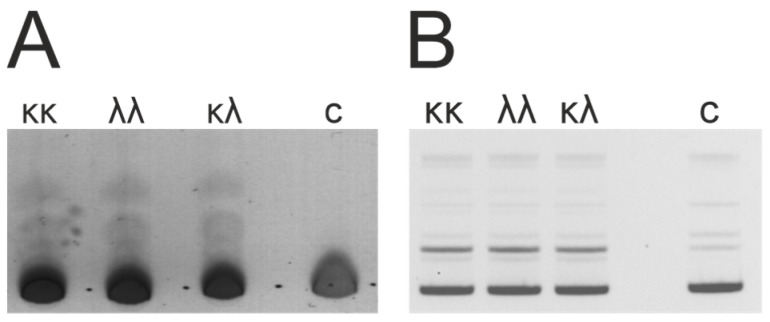
Hydrolysis of maltoheptaose (**A**) and plasmid DNA (**B**) with κκ-, λλ-, and κλ-IgG subfractions in a final concentration of IgG—0.05 mg/mL, incubation time—48 h at 37 °C (typical results for three patients); c—control sample incubated without Abs.

Interestingly, the analysis of both DNA-hydrolyzing (mean ± SD values: 10.9 ± 0.3%; 10.4 ± 0.4%; 10.7 ± 0.3%) and amylolytic activity (mean ± SD values: 7.6 ± 0.5; 7.8 ± 0.3; 7.3 ± 0.4) of κκ-IgG, λλ-IgG, and bispecific κλ-IgG subfractions, respectively, did not reveal any statistically significant differences between the subfractions (Figure 8). Moreover, the average level of DNA-hydrolyzing activity of IgG from 26 patients 8.9 ± 2.6% is very close to the mean values for κκ-IgG, λλ-IgG, and κλ-IgG subfractions. On the contrary, the average level of amylolytic activity of IgG is 4.8 ± 3.0% and is noticeably lower, compared to the subfractions.

### 3.4. Statistical Analysis

Correlation analysis by the non-parametric Spearman rank method did not show statistically significant correlations between the biochemical characteristics of Abs and clinical parameters of patients, except a positive correlation (0.04) between the period of infection and IgG2 content, a negative correlation (−0.03) between the period of infection and IgG1 content, and another negative correlation (−0.04) between the viral load and the level of amylolytic activity. Analysis of the catalytic activity of the κκ-IgG, λλ-IgG, and κλ-IgG subfractions also did not reveal any statistically significant differences. 

## 4. Discussion

Here, we showed how homogeneous IgG preparations were isolated from the blood of 26 HIV-infected patients, from which subfractions of κκ-IgG, λλ-IgG, and bispecific κλ-IgG were isolated using affinity chromatography. It was shown for the first time that the blood of HIV-infected patients contains ~20% of bispecific κλ-IgG, presented with all IgG1–4 subclasses. Since the blood of healthy donors contains ~9% of bispecific κλ-IgG [37], our results may suggest that during the HIV infection, the rate of Fab arms exchange, resulting in a bispecific κλ-IgG generation which may be significantly increased.

A fundamental function of blood Abs is binding to antigen and its neutralization. Furthermore, the antigen is excreted from the body or destructed by cell-mediated immunity [52]. Monospecific Abs are efficient for both of these protective functions. What could be the role of bsAbs, the molecules of which simultaneously contain two different antigen-binding centers? We believe that such Abs can more efficiently cross-link different antigens with each other, and not just interfere with the binding of an infectious agent to cells and reduce the efficiency of infection, but also stimulate Ab-dependent cell-mediated cytotoxicity [53].

For the first time, the ratio of IgG1–IgG4 subclasses was analyzed individually for 26 HIV-infected patients. The IgG1 subclass prevailed in 15 patients while the IgG2 subclass prevailed in others. Three patients with stage 4A and two patients with stage 3 showed a significant predominance of IgG1 subclass (60%), while the dominant subclass for other patients was IgG2 (47%). One may note that the ratio of IgG1–4 subclasses is individual for each patient. 

Analysis of the DNA-hydrolyzing and amylolytic activities of IgG have shown that most of the patients’ Abs hydrolyze plasmid DNA and maltoheptaose. As we know, the analysis of the amylolytic activity of IgG isolate from the blood of HIV-infected patients was described here for the first time, and it is the third type of catalytic activity described for the HIV patients IgGs [54]. Levels of catalytic activity are individual for each patient. We show no statistically significant correlation between the level of DNA hydrolysis, amylolytic activity, HIV subtype, viral load, CD4 count, stage of the HIV infection, and the supposed period of infection.

Natural DNA-hydrolyzing Abs presented in the blood of HIV-infected patients may decrease the concentration of cell-free DNA produced due to apoptosis or other routes, reducing the likelihood of developing autoimmune pathologies and providing a positive effect [22]. In our view, it is unlikely that the amylolytic activity of Abs, hydrolyzing free blood oligosaccharides, has a positive impact on HIV infection. On the other hand, it is known that carbohydrate residues of Env (HIV glycoprotein) may serve as a shield to evade immunity and are targets for broad neutralizing Ab recognition [55]. Although a catalytic Ab decrease in Env glycosylation is very speculative and was not ever shown in vitro or in vivo, we cannot exclude such prospective biological function of natural IgG.

Many papers are devoted to the Abs in HIV infection. Here, we presented the data on the newly diagnosed HIV patients who did not receive antiretroviral therapy. We believe that our data add new facts about natural IgG, including their DNase and amylolytic catalytic activities; the contents of IgG1–IgG4 subclasses; and representation of κκ-IgG, λλ-IgG, and bispecific κλ-IgG. Our future research will focus on possible biological functions of bispecific κλ-IgG and their prospective HIV-neutralizing activity.

Currently, we cannot answer the question regarding whether the blood of HIV-patients contains B-cells, expressing κ and λ light chains on their surface simultaneously, since this also may explain the generation of κλ-IgG, in addition to the Fab arms exchange. It is important to analyze the content of factors stimulating the generation of bsAbs as the result of the exchange between HL fragments (primarily reduced glutathione).

## 5. Conclusions

In this paper, we showed for the first time that the blood of HIV-infected patients contains ~20% of bispecific κλ-IgG, presented with all IgG1–4 subclasses: IgG2 (51.5%) > IgG1 (37.1%) > IgG3 (7.5%) > IgG4 (3.9%). We suppose that these bispecific Abs might be generated due to exchange with HL fragments (Fab arms). We show here for the first time that natural IgG of HIV-infected patients hydrolyzes supercoiled plasmid DNA and oligosaccharide maltoheptaose. These results expand the possible role and biological functions of natural IgG in HIV infection. 

## Figures and Tables

**Table 1:** Patients’ characteristics.

Patient ID	Age	Sex	HIV Subtype	Route of Infection	Year of Infection (Supposed)	Viral Load (Copies per mL)	CD4 Count (Cells per mL)	Stage of HIV ^3^
3	31	male	A6	PWID ^1^	2017	27,700	556	3 ^6^
4	43	male	CRF63_02A	PWID	2017	69,200	568	3
20	38	female	A6	Heterosexual with PWID	2016	75,000	825	3
27	45	female	A6	Het ^2^	2017	31,600	558	2B ^5^
32	38	female	A6	Het	2017	41,000	553	3
39	35	male	A6	PWID	2016	47,800	603	3
41	36	female	A6	Het	2017	84,600	710	3
55	34	female	URF_63/A	PWID	2015	10,500	559	3
92	27	female	URF_63/A	Het	2017	13,100	540	3
96	33	male	A6	PWID	2015	54,700	576	3
98	35	female	A6	PWID	2013	25,700	597	3
99	50	male	A6	PWID	2008	32,900	520	3
101	32	female	CRF63_02A	PWID	2015	120,000	532	3
114	36	male	A6	PWID	2007	87,000	597	3
116	40	male	A6	PWID	2016	90,500	694	3
118	47	male	A6	PWID	2014	85,400	805	3
120	33	female	CRF63_02A	PWID	2014	75,000	600	3
123	28	male	A6	Het	2017	64,500	522	3
126	39	female	URF_63/A	Het	2014	97,800	501	3
134	29	male	A6	Het	2015	no data	962	3
137	32	male	URF_63/A	PWID	2013	175,000	518	4A ^7^
138	39	female	A6	Het	2016	150,000	540	3
140	44	male	A6	PWID	2008	78,400	703	3
142	32	female	A6	Het	2016	19,000	636	3
144	30	male	A6	Het	2016	87,400	530	3
145	33	female	A6	Heterosexual with PWID	2017	34,800	790	2A ^4^

^1^ PWID—persons who inject drugs; ^2^ Het—heterosexual; ^3^ stage of HIV according to the clinical HIV-infection classification; ^4^ 2A—stage of asymptomatic primary manifestations; ^5^ 2B—stage of acute infection without secondary diseases; ^6^ 3—subclinical stage; ^7^ 4A—stage of secondary diseases, as well as bacterial, fungal, and viral lesions of mucous membranes and the skin, and inflammatory diseases of the upper respiratory tract.

## Data Availability

Most of the relevant raw experimental results are given in the Supplementary Section. Other empirical data that do not relate to the personal data of HIV-infected patients can be provided by request to Anna Timofeeva.

## Supplementary Materials

The following supporting information can be downloaded at: https://www.mdpi.com/article/10.3390/life12020304/s1, Table S1: Double sandwich ELISA of affinity isolated IgG subfractions (κκ-IgG, λλ-IgG, κλ-IgG). Primary antibodies-anti-kappa-light chains and anti-lambda-light chains. Detection with anti-kappa and anti-lambda conjugates with horseradish peroxidase. Data series of three independent experiments is presented in the Table 1. The experimental error did not exceed 5%; Table S2: Ratio of subclasses in IgG preparations isolated from the blood of HIV-infected donors. The analysis was performed by double sandwich ELISA. Primary anti-IgG1–IgG4 antibodies were sorbed in 96-wells ELISA plate. Detection was carried out using anti-IgG-HRP conjugates. Data from a series of three independent experiments are presented in the table; the experimental error did not exceed 5%; Table S3: ELISA analysis of IgG subclasses in subfractions of λλ-IgG, κκ-IgG, κλ-IgG isolated form the blood of HIV-infected donors. The analysis was performed by double sandwich ELISA as in Table S2; Table S4: Relative catalytic activities of IgG preparations isolated from the blood plasma os 26 HIV-infected patients. Hydrolysis of plasmid DNA pBluescript (final concentration 9.6 μg/mL) and maltoheptaose (final concentration 5.0 mM) by individual IgG preparations. Incubation for 24 h at 37 °C. Data from a series of three independent experiments presented in Table, the experimental error did not exceed 5%; Figure S1: Original images for Figure 1; Figure S2: Original images for Figure 5.

## Appendix A

Results of in situ DNase activity (Figure A1A) show that the hydrolysis of calf thymus DNA co-polymerized in polyacrylamide gel occurs in a single zone (Figure A1A, line 2), corresponding to the IgG band (Figure A1A, line 1). Amylolytic activity was investigated using the fragments of gel sliced to the numerous fragments from high molecular mass to low molecular masses (Figure A1C). As one can see from Figure A1B, visible hydrolysis of maltoheptaose was detected only in the gel fragments, corresponding to the IgG band (Figure A1C).

Like DNase I or alpha-amylase, canonical enzymes provide high catalytic activity in a short time of incubation; catalytic Abs possess much lower activities. In Figure A2, we present the results of concentration-dependent activity of canonical DNA- and oligosaccharide-hydrolyzing enzymes—DNase I and alpha-amylase.

In Table A1, we present the results of conversion DNase and amylolytic catalytic activities of HIV patients IgGs in enzyme units, corresponding to the commercial DNase I and α-amylase. Using the calibration curve of enzyme activities (DNase I and α-amylase), we calculated the specific DNA-hydrolyzing and amylolytic activity of IgG samples (Table A1).

**Table A1:** Conversion of IgG catalytic activity to the enzyme activity units corresponding to the DNase I and α-amylase.

Patient ID	DNase Activity, U/mg of IgG	Amylolytic Activity, U/mg of IgG
3	6.6 × 10^−16^	2.2 × 10^−8^
20	3.3 × 10^−16^	9.2 × 10^−9^
32	0.8 × 10^−16^	9.9 × 10^−9^
39	1.1 × 10^−15^	9.3 × 10^−9^
55	1.1 × 10^−13^	1.4 × 10^−8^
92	3.5 × 10^−17^	1.7 × 10^−8^
98	2.8 × 10^−18^	1.3 × 10^−8^
99	0.7 × 10^−16^	0
114	3.0 × 10^−16^	1.4 × 10^−8^
118	1.6 × 10^−15^	1.2 × 10^−8^
123	0.6 × 10^−16^	1.5 × 10^−8^
126	0.6 × 10^−16^	9.1 × 10^−9^
137	1.1 × 10^−17^	1.1 × 10^−8^
138	1.6 × 10^−16^	0
140	0.6 × 10^−17^	8.5 × 10^−9^
142	4.6 × 10^−17^	8.0 × 10^−9^
144	0.6 × 10^−16^	7.5 × 10^−9^
145	1.0 × 10^−17^	8.5 × 10^−9^
4	2.0 × 10^−19^	2.6 × 10^−8^
27	1.0 × 10^−16^	1.0 × 10^−8^
41	4.8 × 10^−16^	1.6 × 10^−8^
96	1.6 × 10^−17^	1.2 × 10^−8^
101	1.0 × 10^−17^	3.0 × 10^−8^
116	1.0 × 10^−16^	1.6 × 10^−8^
120	1.0 × 10^−16^	9.3 × 10^−9^
134	1.2 × 10^−16^	1.1 × 10^−8^

As one can see from Table A1, the differences in specific DNase activity varies in six orders of magnitude: from 10^−19^ to 10^−13^ U/mg of IgG, depending on the patient ID. On the contrary, the detectable amylolytic activity ranges in less than two orders: from 10^−9^ to 10^−8^ U/mg.

EDTA is one of the non-specific inhibitors of metal-dependent DNases and enzymes which hydrolyze sugars. We used EDTA in concentrations 1 to 10 mM to show the inhibition of these catalytic activities (Figure A3).
