# Supplementary material for: The Blood of the HIV-Infected Patients Contains κ-IgG, λ-IgG, and Bispecific κλ-IgG, Which Possess DNase and Amylolytic Activity
Source: Life (Basel). 2022 Feb 17;12(2):304. doi: 10.3390/life12020304 (PMC8880267; doi:10.3390/life12020304)

Table S1. Double sandwich ELISA of affinity isolated IgG subfractions (κκ-IgG, λλ-IgG, κλ-IgG). Primary antibodies - anti-kappa-light chains and anti-lambda-light chains. Detection with anti-kappa and anti-lambda conjugates with horseradish peroxidase. Data series of three independent experiments is presented in the Table 1. The experimental error did not exceed 5%.

		A_{450} Values		
**Primary Antibody**	**Detection Antibody**	**κκ-IgG**	**λλ-IgG**	**κλ-IgG**
**anti-kappa-light chain**	**anti-kappa-light chain HRP**	1,415	0,053	0,069
	**anti-lambda-light chain HRP**	0,043	0,046	1,100
**anti-lambda-light chain**	**anti-lambda-light chain HRP**	0,044	1,452	0,081
	**anti-kappa-light chain HRP**	0,037	0,049	1,298

Table S2. Ratio of subclasses in IgG preparations isolated from the blood of HIV-infected donors. The analysis was performed by double sandwich ELISA. Primary anti-IgG1-IgG4 antibodies were sorbed in 96-wells ELISA plate. Detection was carried out using anti-IgG-HRP conjugates. Data from a series of three independent experiments are presented in the table; the experimental error did not exceed 5%.

Donor ID	IgG1, %	IgG2, %	IgG3, %	IgG4, %
4	42.3	34.2	16.3	7.1
27	29.7	51.8	12.7	5.9
41	52.8	34.2	9.9	3.1
96	36.6	46.0	11.6	5.8
101	41.2	40.0	11.5	7.3
116	45.7	38.5	9.9	5.9
120	45.3	38.2	13.1	3.5
134	49.5	30.4	12.5	7.6
138	34.5	45.4	14.9	5.2
142	32.1	50.1	15.5	2.4
144	35.3	49.9	11.5	3.3
145	32.3	47.6	14.0	6.2
3	39.0	41.5	12.5	7.0
20	57.2	26.3	13.9	2.6
32	57.6	30.3	8.2	3.9
39	59.9	18.1	12.9	9.1
55	43.8	37.8	13.3	5.2
92	35.2	44.8	15.0	5.0
99	49.3	30.0	15.6	5.2
114	60.5	21.7	13.7	4.0
118	41.0	42.9	10.5	5.6
123	38.0	44.9	11.9	5.2
126	38.1	46.4	12.4	3.1
137	59.5	20.5	12.4	7.5
140	35.4	45.0	15.7	3.9
98	41.5	39.9	11.0	7.7

Table S3. ELISA analysis of IgG subclasses in subfractions of λλ-IgG, κκ-IgG, κλ-IgG isolated form the blood of HIV-infected donors. The analysis was performed by double sandwich ELISA as in Table S2.

	IgG1, %	IgG2, %	IgG3, %	IgG4, %
**κκ-IgG**	**55,8**	**34,2**	**7,4**	**2,6**
**λλ-IgG**	**57,1**	**35,5**	**5**	**2,3**
**κλ-IgG**	**37,1**	**51,5**	**7,5**	**3,9**

Table S4. Relative catalytic activities of IgG preparations isolated from the blood plasma os 26 HIV-infected patients. Hydrolysis of plasmid DNA pBluescript (final concentration 9.6 μg/ml) and malto-heptaose (final concentration 5.0 mM) by individual IgG preparations. Incubation for 24 h at 37 °C. Data from a series of three independent experiments presented in Table, the experimental error did not exceed 5%.

Donor ID	DNase Activity, %	Amylolytic Activity, %
3	10.6	9.4
20	10.5	3.1
32	8.9	3.6
39	11.8	3.2
55	16.7	6.1
92	8.1	7.8
98	5.4	5.7
99	8.8	0.2
114	10.4	6.3
118	12.2	5.3
123	8.6	6.8
126	8.7	3.0
137	6.9	4.1
138	9.7	0.0
140	6.1	2.5
142	8.4	2.1
144	8.7	1.6
145	6.8	2.5
4	2.6	10.7
27	9.2	3.8
41	10.9	7.0
96	7.3	4.8
101	6.8	11.7
116	9.2	7.1
120	9.2	3.2
134	9.4	4.4

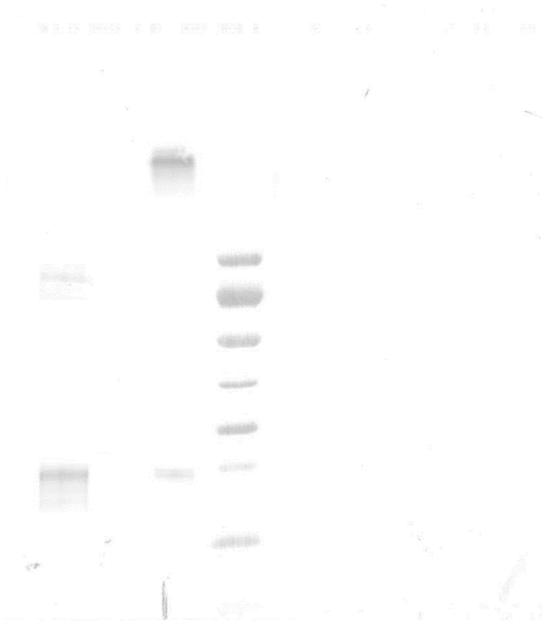

Figure S1. Original images for Figure 1.

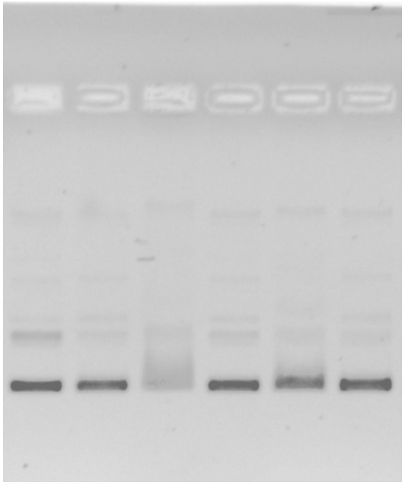
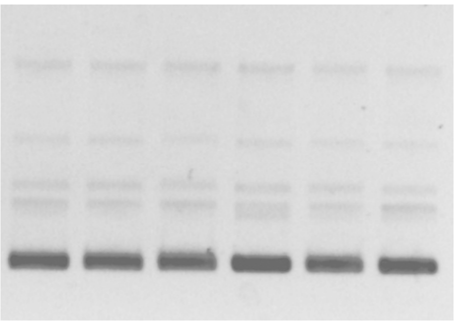
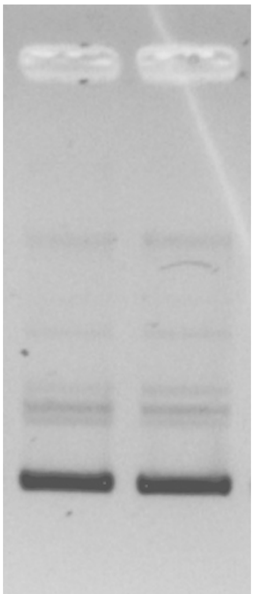

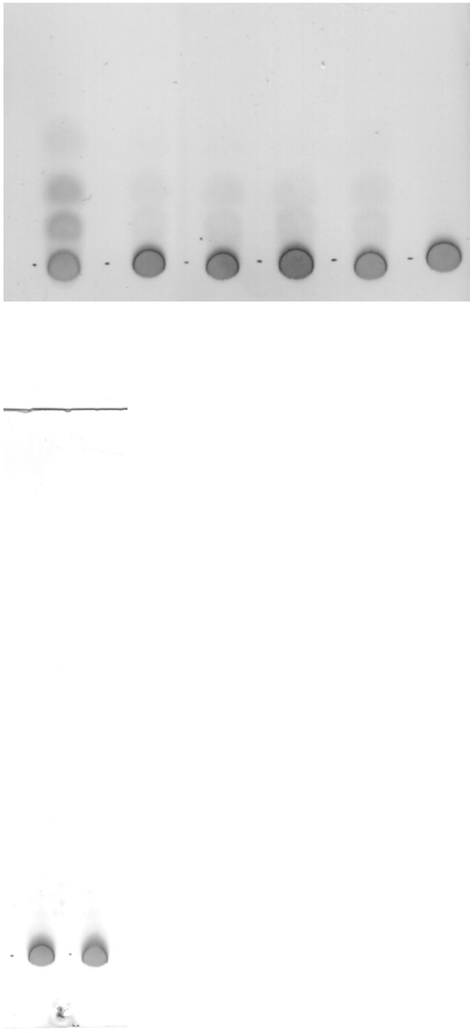

Figure S2. Original images for Figure 5.

Original images for **Figure 6**

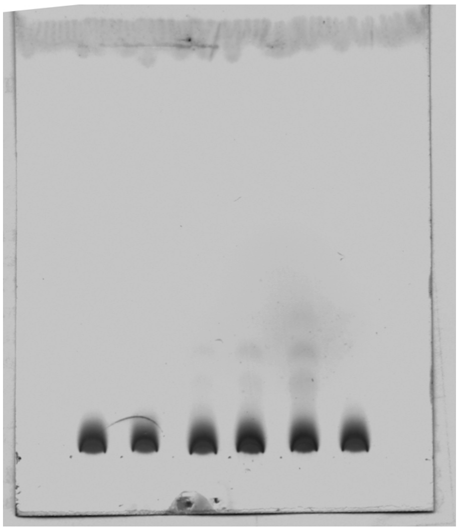

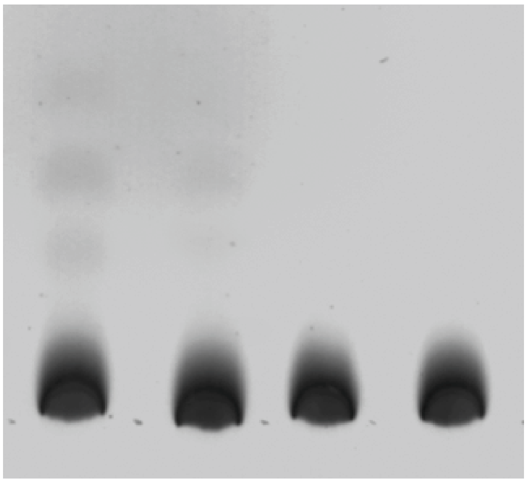

Original images for **Figure 8**

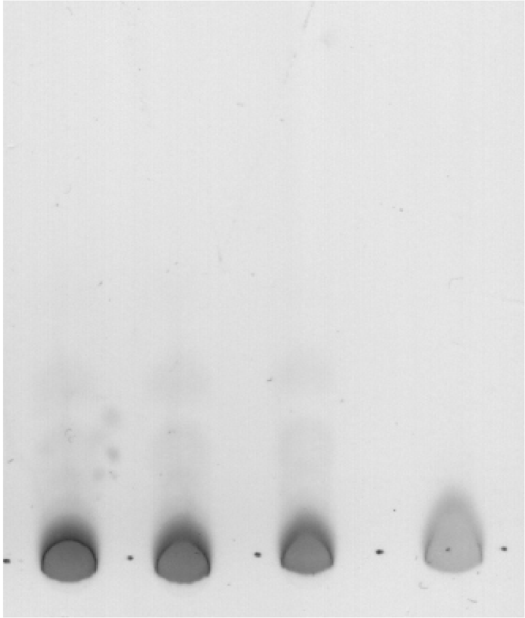

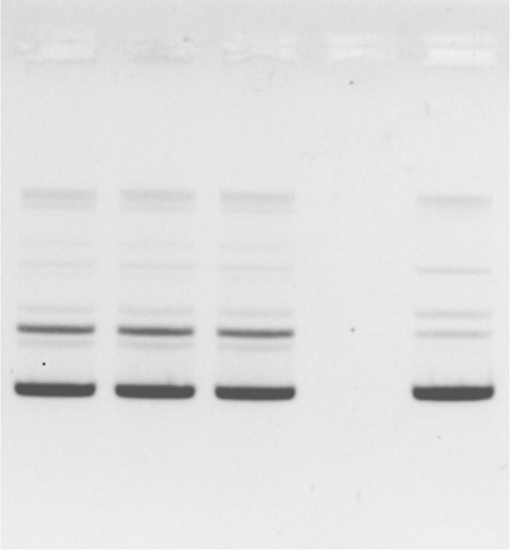

Supplement: Supplementary file 1 [file life-12-00304-s001.zip › life-1570931-supplementary.pdf]
